# Delay in the Detrended Fluctuation Analysis Crossover Point as a Risk Factor for Type 2 Diabetes Mellitus

**DOI:** 10.1155/2016/9361958

**Published:** 2016-05-16

**Authors:** Manuel Varela, Luis Vigil, Carmen Rodriguez, Borja Vargas, Rafael García-Carretero

**Affiliations:** ^1^Servicio de Medicina Interna, Hospital Universitario de Mostoles, Rio Jucar s/n, Mostoles, 28935 Madrid, Spain; ^2^European University of Madrid, Villaviciosa de Odón, Spain

## Abstract

Detrended Fluctuation Analysis (DFA) measures the complexity of a glucose time series obtained by means of a Continuous Glucose Monitoring System (CGMS) and has proven to be a sensitive marker of glucoregulatory dysfunction. Furthermore, some authors have observed a crossover point in the DFA, signalling a change of dynamics, arguably dependent on the beta-insular function. We investigate whether the characteristics of this crossover point have any influence on the risk of developing type 2 diabetes mellitus (T2DM). To this end we recruited 206 patients at increased risk of T2DM (because of obesity, essential hypertension, or a first-degree relative with T2DM). A CGMS time series was obtained, from which the DFA and the crossover point were calculated. Patients were then followed up every 6 months for a mean of 17.5 months, controlling for the appearance of T2DM diagnostic criteria. The time to crossover point was a significant predictor risk of developing T2DM, even after adjusting for other variables. The angle of the crossover was not predictive by itself but became significantly protective when the model also considered the crossover point. In summary, both a delay and a blunting of the crossover point predict the development of T2DM.

## 1. Introduction

Glycaemic variability is considered a risk factor for diabetic complications, over and above raw glycaemic levels (as measured through fasting blood glucose or glycosylated haemoglobin) [1–3]. However, there is still controversy about which metric should be used to assess these dynamic aspects [4]. Conventional statistics (standard deviation, coefficient of variability) have the pitfall of considering every measure as independent, thus overlooking an essential part of the time series: its sequentiality. Mean Amplitude of Glycaemic Excursions (MAGE) takes sequentiality into account but fixes an arbitrary threshold of “significant” excursions, thus overlooking the fine-grain regulation.

Complexity analysis of glucose time series, measured by means of Detrended Fluctuation Analysis (DFA), has emerged as a useful alternative and is increasingly being used as a standard to measure glucose dynamics, especially in diabetic patients [5–15]. In all of these papers, there is a consistent correlation between loss of complexity (i.e., increased DFA) and glucoregulatory dysfunction.

Ogata et al. [9] have also described a crossover point in DFA, located approximately in the 2-hour time window. Furthermore, they observed a decrease in long-range negative correlations (i.e., decreased complexity in large time windows) in patients with diabetes [11]. Although DFA has mainly been used in patients with diabetes, several papers suggest that there is a progressive fall in complexity (i.e., increase in DFA) as a patient walks his way from health, through the prediabetes states to full-blown type 2 diabetes mellitus (T2DM) [6, 9–12, 15–17].

The present study intends to analyse the characteristics of the DFA crossover point in a population with high risk of becoming diabetic and to find out if these characteristics may have any influence on the risk of developing T2DM.

## 2. Methods

### 2.1. Patients

A sample of 262 patients from the Internal Medicine Outpatient Clinic and the Vascular Risk Unit of the University Hospital of Mostoles were selected based on an assumed increased risk of developing T2DM. The main characteristics of this sample have been published previously [12]. The inclusion criteria were an HbA1c > 5% and <6.5% and any of the following:

- essential hypertension;
- BMI ≥ 30 Kg/m^2^;
- a first-degree relative with a diagnosis of T2DM.

Patients were excluded if they had a diagnosis of DM or were on drugs that could interfere with glucose regulation (e.g., glucocorticoids).

After an interview, physical exam, and routine biochemical tests, a 3-day glucometry was performed by means of a Continuous Glucose Monitoring System device (iPro, Medtronic MiniMed, Northridge, CA, USA). The glucometry was obtained in an ambulatory setting, while the patient followed his normal life, with no special dietary restrictions. The patient was thereafter followed up every 6 months with a clinical visit and routine biochemical tests. The present study is an interim analysis on the project's third year.

The main outcome was a diagnosis of T2DM (basal glycaemia ≥ 7.0 mmol/L, glycosylated haemoglobin (HbA1c) ≥ 6.5%, or starting on antidiabetic drugs).

### 2.2. DFA and Crossover

From the glucometry obtained at admission, a clean, 24-hour-long time series was selected for each patient. Whenever possible, the selected 24-hour sequence started at 08.00 AM the day after the device insertion, to avoid the stressful hours in the hospital. If there were missing values, these were obtained by interpolation as long as the missing string was <3 consecutive values. If there were three or more consecutive missing values, another 24-hour period was selected. If no adequate 24-hour period was found, the time series was considered unsuitable and discarded.

Each selected series was thus composed of 288 consecutive measures of interstitial glucose, sampled every 5′.

Each time series was submitted to Detrended Fluctuation Analysis, without previous integration. A full description of DFA may be consulted in [18]. A brief description can be found in [12], and a basic introductory video is available at http://www.complexity-at-the-bedside.org/complexity/tutorials/.

**Figure 1:**
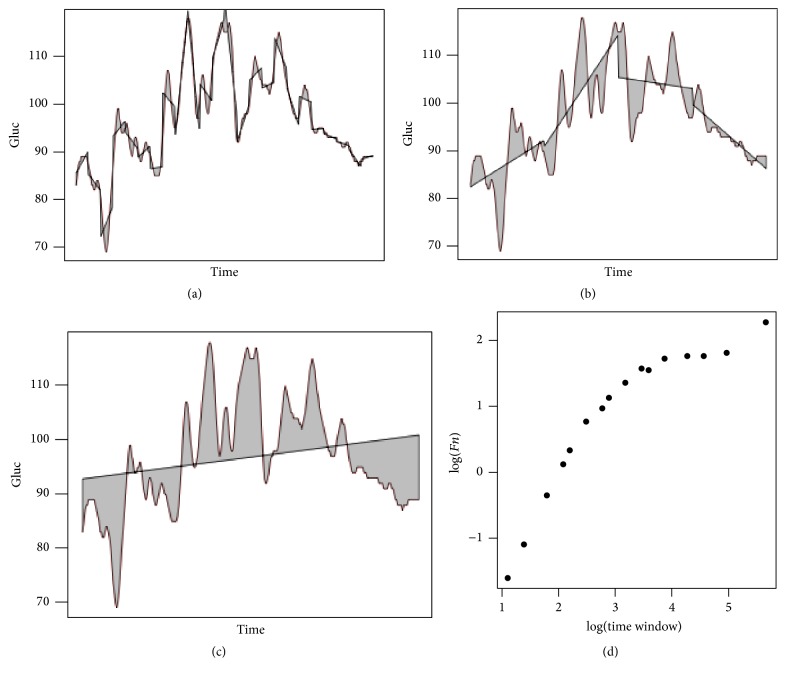
DFA analyses how the time series (the “territory”) and its representation through linear regression (the “map”) diverge as the time window considered increases. (a), (b), and (c) display this “territory versus map gap” (grey area) with three different time windows. (a) One-hour time window (12 points in each regression line). (b) Six-hour time window (36 points in each regression line). (c) Twenty-four-hour time window (288 points in the regression line). The complete windowing sequence used was 3, 4, 6, 8, 9, 12, 16, 18, 24, 32, 36, 48, 72, 96, 144, and 288 points (corresponding to time windows of 15′, 20′, 30′, 40′, 45′, 60′, 80′ 90′, 120′, 160′, 180′, 240′, 360′, 480′, 720′, and 1440′). (d) plots the log(“map-to-territory gap”) versus log(time window). The slope of a regression line through this set of points (not shown) would be the DFA of the time series (not considering the crossover).

In essence, DFA estimates the degree of long-range correlations within a signal, analysing how the time series and its linear regression diverge as the “time window” considered increases (Figure 1). Metaphorically, one could consider the linear regression of each time window as a “map” of a certain “territory.” As the time windows increase, the regression's fitness deteriorates, and thus the “map-to-territory gap” increases. The rate at which this gap increases reflects how the informational content of the time series is distributed. A high-complexity time series will have comparatively more information encoded in the small windows. Conversely, low-complexity time series will have more information encoded in the large time windows, and therefore the “map-to-territory gap” will be increasing at a steady pace well into larger time windows.

Specifically, we submitted the time series (without pretreatment by integration) to detrending with a windowing sequence of 3, 4, 6, 8, 9, 12, 16, 18, 24, 32, 36, 48, 72, 96, 144, and 288 points (corresponding to time windows of 15′, 20′, 30′, 40′, 45′, 60′, 80′, 90′, 120′, 160′, 180′, 240′, 360′, 480′, 720′, and 1440′).

For each time window, a “map-to-territory gap” was calculated:

(1) $$\begin{array}{c} Fn=\sqrt{\frac{1}{N}\overset{N}{\underset{k=1}{\sum }}{\left[y\left(k\right)-y_{n}\left(k\right)\right]}^{2}}. \end{array}$$

A log⁡(*Fn*) ~ log(time window) was drawn for each glucometry, with 16 points (the aforementioned time windows).

**Figure 2:**
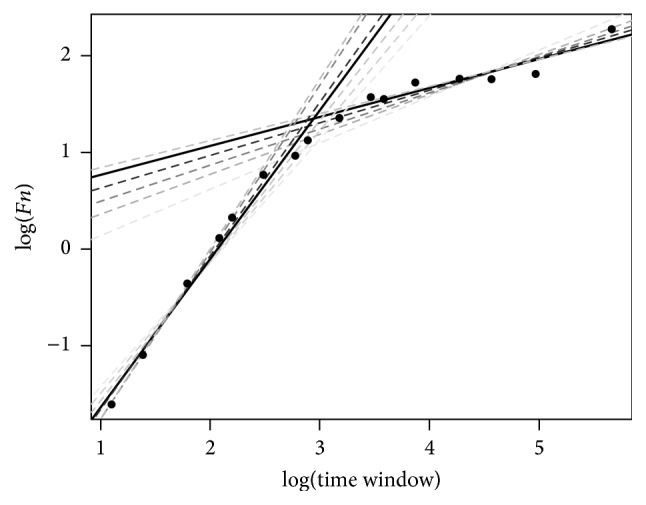
To calculate the crossover point, a set of pairs of linear regression lines are built with several combinations of points: points 1–4 for the first limb and 5 : 16 for the second, then 1 : 5 and 6 : 16, then 1 : 6 and 1 : 16, and so on until 1 : 11 and 12 : 16. A combined *R* ^2^ is calculated for each pair of regression lines, and the best-fit pair is assumed to be the best representation of the time series. In this figure, the shade of the regression lines represents the goodness of fit (darker grey: better fit). The best fit is represented by a solid line. The abscissa of the intersection between both limbs is the crossover point (represented as log(number of measurements per window)). To obtain the time (in minutes) for a value *x*, crossover (minutes) = *e* ^(5·*x*)^.

Next, a set of pairs of linear regressions was built for several combinations of points (i.e., points 1–4 for the first limb and 5–16 for the second, then 1–5 and 6–16, then 1–6 and 7–16, etc., until 1–11 and 12–16) (Figure 2).

A combined weighted *R* ^2^ was obtained for each pair of regression lines, and the best-fit pair was selected as the best representation of the time series. The abscissa of the intersection of both limbs, expressed in minutes, was considered the crossover point, and the angle was from the difference between the slopes of the two limbs. The slope of the first and second limb was assumed to be the DFA for the short and long time windows, respectively.

### 2.3. Statistical Analysis

Comparison between admitted and excluded patients was performed by means of *t*-test (for quantitative variables) or Chi-square test (for qualitative variables).

The effect of the various variables was analysed by means of a multivariate Cox proportional hazard survival analysis. The statistical analysis was performed in R (R (http://www.r-project.org/)). Significance was set at two-tailed *p* < 0.05, although *p* < 0.10 were also displayed.

## 3. Results

Of the 262 patients initially included, 40 were finally excluded because we were not able to obtain a suitable glucometry. 15 patients had no follow-up visits, and one patient was excluded because she started on high-dose glucocorticoids due to a facial palsy. Except for a slightly lower diastolic blood pressure (73.9 mmHg versus 78.1 mmHg, *p* = 0.01), there were no major differences between admitted and excluded patients regarding anthropometric, physical exam or analytical parameters. Thus, exclusion did not seem to carry any bias.

The 206 patients finally included were followed up for a mean of 18 months (IQR 15) (Table 1). There were 18 events (T2DM new diagnoses), for an incidence of 58.2 cases/1000 patients-year.

The median to the crossover point was 114 min (IQR 64.7 min), and the median angle between the first limb (small time windows, before the crossover point) and the second limb (large time windows) was 0.64 radians (IQR 0.17 rad).

In a Cox proportional hazard ratio model, the crossover point was a significant risk factor for the development of T2DM (*β* = 0.015, *p* < 0.001). This implied a hazard rate of 1.53 for every 30-minute delay in crossover. These results did not change significantly when adjusting for other relevant variables, whether anthropometric (gender, age, body mass index, and waist circumference), clinical (blood pressure and first-degree relatives with diabetes), or analytical (HbA1c, insulin, mean glucose, glucose standard deviation, MAGE, HOMA, or global DFA). When adjusting for basal glycaemia, the effect of crossover did not attain significance, although it persisted as a trend (*p* = 0.08).

The crossover angle had no significant influence on the development of T2DM when considered alone (*β* = −2.43, *p* = 0.15) but became significantly protective when the model considered also the crossover point (*β* = −4.172, *p* = 0.005). Similarly, neither the DFA of the first nor the second limb (before and after the crossover) alone had significant influence on the hazard rate of developing T2DM (dfa1: *β* = −0.475, *p* = 0.735; dfa2: *β* = 1.452, *p* = 0.279), but they both became significant when adjusting for the crossover point (dfa1: *β* = 4.876, *p* = 0.018; dfa2: *β* = 4.050, *p* < 0.001).

## 4. Discussion

DFA analyses how the correlation between successive points evolve as the time-window considered increases. Following the conventional homeostatic paradigm, a healthy physiological system should promptly detect trends and unleash mechanisms to correct them. Therefore, it is reasonable to expect a deterioration in the fit of the regression line as the time windows increase. Furthermore, one would expect that as the physiological system becomes old or dysfunctional, its response will become “sluggish,” and the decorrelation will be slower. This loss of sharpness (i.e., loss of complexity) is displayed as an increase in DFA. Indeed, there is ample evidence relating diabetes mellitus with an increase in glucose time series' DFA [6, 7, 9–12, 16].

An important advantage of DFA with respect to other conventional dynamic metrics (i.e., coefficient of variability or MAGE) is that it considers the time series as a whole, not as a set of independent measurements (as with the coefficient of variability) nor does it make any assumptions on the “significance” of each glycaemic excursion (as with MAGE).

Glucoregulation is a rather asymmetric system: while there are at least four main counterregulatory hormonal systems in charge of fighting hypoglycaemia (glucagon, alpha-sympathomimetics, glucocorticoids, and growth hormone), there is only one strictly antihyperglycaemic hormone, namely, insulin. This has obvious evolutionary justifications (short-term hypoglycaemia is far more dangerous than hyperglycaemia) but may cause significant differences in the counterregulatory dynamics. While the hyperglycaemic drift may be a swift, multisystem driven reaction, the antihyperglycaemic push is mainly a one-man job and may therefore have more abrupt characteristics. Arguably this may explain the dynamic change underlying the crossover point described by Ogata et al. [9]. If this were the case, it would be reasonable to expect a progressive delay (and fading) of this dynamic change as the beta-function deteriorates, long before its failure allows for the diagnosis of diabetes.

Our findings of a delay in the crossover point and a blunting of the angle between both limbs as prognostic factors for the development of T2DM in patients at increased risk are congruent with this hypothesis. Arguably, this may represent both a delay and a dampening of the insulin kick-in and may reveal an early dysfunction of glucoregulation. This should be further confirmed by means of conventional beta-function examination. However, our model has significant advantages over other experimental evaluations of beta-function: it may be applied in real-life situations rather than in the laboratory, it is much simpler, and it displays the functioning of the glucoregulatory system as a whole, not as the specific response to a certain glycaemic load or insulin infusion.

We may be starting to have drugs available that can delay or prevent the evolution to T2DM in subjects at risk [19–23]. It will be crucial to identify those patients who would eventually walk all their way to diabetes in order to better target therapeutic interventions. Classic variables (basal glycaemia, oral glucose tolerance test, and HbA1c) are probably insufficient, and it is not through fine-tuning thresholds that this problem will be solved. Arguably, glucodynamic techniques studying how glucose levels fluctuate in time may afford a fresh, new insight into this problem.

**Figure 3:**
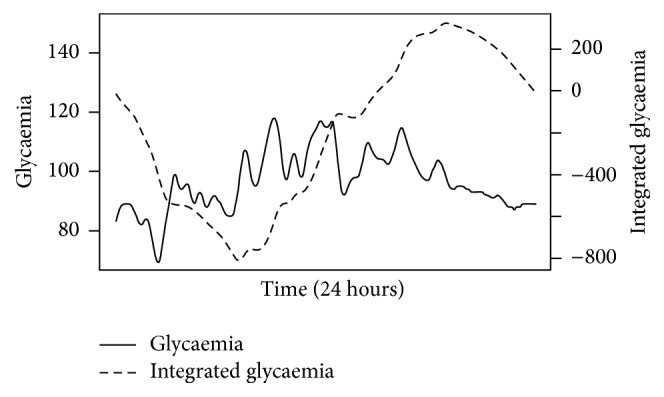
Glycaemia (solid line, left axis) and integrated glycaemia (dashed line, right axis). Generally, before proceeding to the detrending process mentioned in Figure 1, most authors preprocess the time series through integration: *y*(*k*) = ∑_*i*=1_^*k*^(*G*_*i*_ − *G*_mean_), where *y*(*k*) is the integrated value, *G*_*i*_ is each individual measurement, and *G*_mean_ is the mean of the series. The resulting integrated time series complies with the conventional random-walk model and thus is easier to interpret. However, this standardization comes at the price of a significant smoothing of the time-series profile, thus arguably loosing significant information.

It should be mentioned that, contrary to most studies with DFA in glycaemia, we have not preprocessed the time series through integration before performing the sliding-windows fluctuation analysis. This arguably takes us out of the conventional random-walk model and the standard 1.5 threshold of “brown” noise (integrated random series) DFA cannot be applied. Our model is therefore only a tool to compare different time series (within similar series length and time windows). However, integrating the time series erases important information (e.g., Figure 3 displays the same time series, with and without pretreatment through integration), and we believe that preserving this information is worth the loss of standardization caused by omitting the conversion to a random-walk model. DFA measures the complexity of a time series by evaluating how the “map-territory gap” enlarges (i.e., how the linear regression and the curve diverge) as the time window increases and thus provides a useful measure of the series' entropy even omitting the random-walk model.

We have run the same analysis before treating the time series through integration, and although the same tendencies persist, the crossover effects are much less obvious and often do not reach statistical significance.

### 4.1. Study Limitations

Oral glucose tolerance tests were not performed, and thus neither Impaired Glucose Tolerance nor insulin response to OGTT could be analysed.

The notion that a delay in crossover represents a dampening of beta-function is only a hypothesis and needs confirmation through conventional experimental tests.

## 5. Conclusions

The characteristics of the crossover phenomenon have predictive value for the development of T2DM in patients at risk and may provide a sensitive and easy way to explore the earliest signs of glucoregulatory failure.

## Figures and Tables

**Table 1:** Patients' characteristics.

History and physical exam	
Age (years) (median, IQR)	61 (13)
Gender (F/M)	101/105
Smoking habit (%)	23 (11%)
Relatives with T2DM (%)	55 (28%)
Systolic BP (mmHg) (median, IQR)	133.5 (19.25)
Diastolic BP (mmHg) (mean, SD)	78.2 (9.0)
BMI (Kg/m^2^) (median, IQR)	30 (6)
Abdominal perimeter (cm) (mean, SD)	
Men	104.5 (10.1)
Women	99.2 (12.1)

Complementary tests	

Basal glycaemia (mmol/L) (mean, SD)	5.56 (0.62)
HbA1c (%) (median, IQR)	5.76 (0.3)
IFG (%)	105 (51%)
HbA1c ≥ 38.3 mmol/mol (%)	129 (66%)
HDL-cholesterol (median, IQR)	
Men	1.35 (0.35)
Women	1.50 (0.32)
Triglycerides (mmol/L) (median, IQR)	0.125 (0.71)
EPI-GFR (mL/min/1.73 m^2^) (mean, SD)	93.0 (9.5)
Insulin (mlU/L) (median, IQR)	11.7 (9.5)
HOMA-index (median, IQR)	3.06 (2.27)
Albuminuria (mg/gr creatinine) (median, IQR)	2.78 (6.15)
Number of ATP-III MS defining criteria (median, IQR)	2 (1)
Number of patients complying with the ATP-III MS definition (≥3 criteria)	100 (49%)

Glucometry	

Median glucose of the time series (median, IRQ)	5.44 (0.89)
Median SD of the time series (median, IRQ)	0.81 (0.41)
CV (%) glucose time series (median, IQR)	14.2 (6.7)
MAGE (mg/dL) (median, IQR)	36.5 (22.9)
DFA (whole time series) (mean, SD)	0.90 (0.09)

Crossover	

Time to crossover (min) (mean, IQR)	114.0 (64.7)
Crossover angle (rad) (mean, IQR)	0.64 (0.17)
DFA first limb (mean, IQR)	1.53 (0.23)
DFA second limb (mean, IQR)	0.36 (0.24)

T2DM: type 2 diabetes mellitus; BP: blood pressure; BMI: body mass index; IFG: impaired fasting glucose (basal glucose ≥ 100 mg/dL); EPI-GFR: estimated glomerular filtration rate (EPI-creatinine equation); HOMA: homeostasis model assessment; MS: metabolic syndrome; CV: coefficient variation; MAGE: mean average glucose excursions; DFA: Detrended Fluctuation Analysis.

Mean and standard deviation for normally distributed variables and median and interquartile range for nonnormally distributed variables.
